# Supplementary material for: A circuit from the ventral subiculum to anterior hypothalamic nucleus GABAergic neurons essential for anxiety-like behavioral avoidance
Source: Nat Commun. 2022 Dec 3;13:7464. doi: 10.1038/s41467-022-35211-7 (PMC9719513; doi:10.1038/s41467-022-35211-7)
Supplement: Supplementary file 1 — Supplementary Information [file 41467_2022_35211_MOESM1_ESM.pdf]

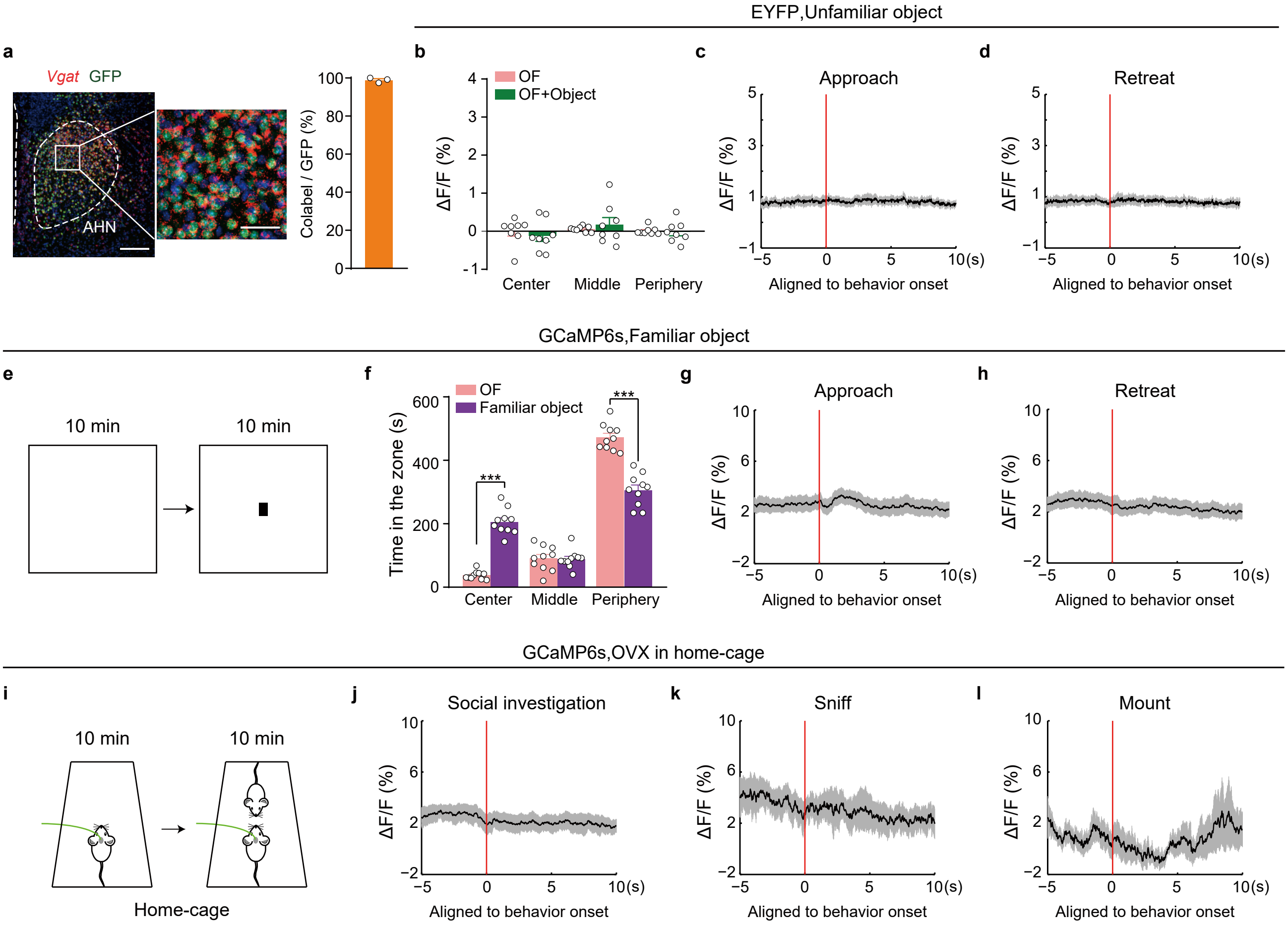

Supplementary Figure 1. Verification of the *Vgat-IRES-Cre* mouse line and fiber photometry recordings of AHN^{Vgat+} neurons expressing EYFP or GCaMP6s.

a AAV-EF1α-DIO-H2B-EGFP was injected into AHN of *Vgat-IRES-Cre* males. A representative image on the left shows *Vgat* signals and viral-mediated GFP expression in AHN. Scale bar, 200 μm. The magnified image on the right highlights the area within the white box. Scale bar, 50 μm. Quantification of the co-localization of *Vgat* and GFP signals. n = 3 mice. **b-d** Fiber photometry recordings of EYFP mice in open field with an unfamiliar object. n = 8 mice. Wilcoxon matched-pairs signed rank test for "center" and "periphery" signal, and two-tailed paired t test for "middle" signal. **e-h** Fiber photometry recordings of GCaMP6s males with a familiarized object. n = 10 mice. (e) The object (a battery) used was placed in the mouse's homecage for three days before introduced to the open field. (f) Quantification of the time the mice spent in each zone of the open field before or after introduction of the familiar object. Mice spent significant time in the center zone after object introduction. Two-tailed paired t test. Center, $p < 0.0001$, middle, $p = 0.86$, periphery, $p < 0.0001$. (g-h) Average values of GCaMP6s ΔF/F signal aligned to approach (g) or retreat onset (h) at the time "0". No changes in AHN^{Vgat+} activity was detected during either behavior. **i-l** Fiber photometry recordings of GCaMP6s males interacting with a novel, hormonally primed ovariectomized (OVX) female mouse in the home cage. n = 9 mice. (i) Schematics of the behavioral protocol. No changes in AHN^{Vgat+} activity was detected during social investigation (j), sniff (k), or mount (l). ***, $p < 0.001$. Data are presented as mean values +/- SEM. In panel (c)-(d), (g)-(h), (j)-(l), the solid line inindicates the mean and the shaded area is the SEM.

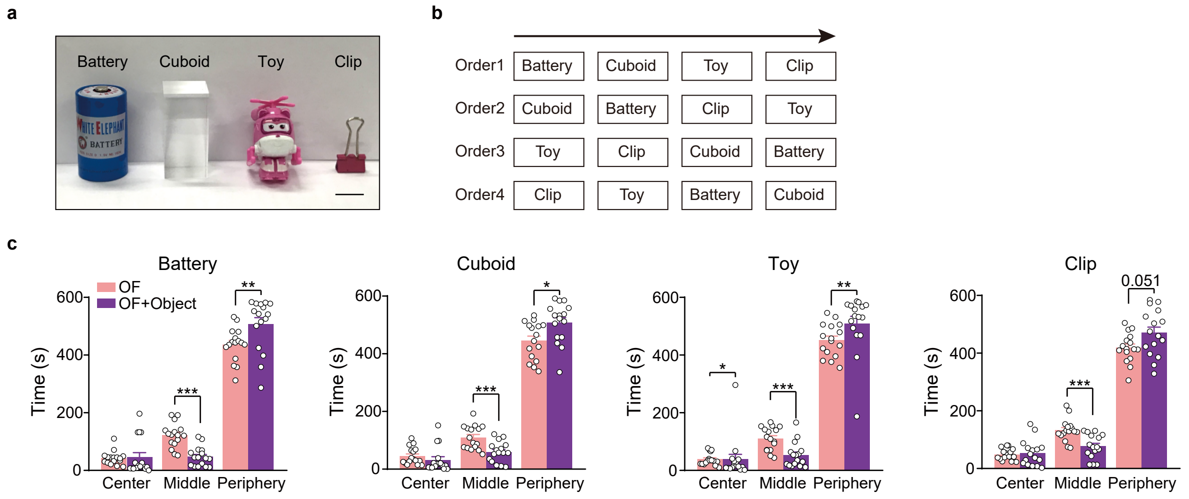

Supplementary Figure 2. Different objects induced similar center avoidance and periphery preference in the open field test.
(**a**) Different unfamiliar objects used. (**b**) The order in which the unfamiliar objects were presented on separate testing days. (**c**) Time spent in the center, middle, and periphery zone of the open field before or after the indicated object was introduced. n = 16 mice. battery, center, Wilcoxon matched-pairs signed rank test, $p = 0.67$, middle, two-tailed paired t test, $p < 0.0001$, periphery, Wilcoxon matched-pairs signed rank test, $p = 0.005$; cuboid, center, Wilcoxon matched-pairs signed rank test, $p = 0.18$, middle, two-tailed paired t test, $p = 0.0004$, periphery, two-tailed paired t test, $p = 0.02$; toy, Wilcoxon matched-pairs signed rank test, center, $p = 0.04$, middle $p < 0.0001$, periphery, $p = 0.005$; clip, two-tailed paired t test, center, $p = 0.75$, middle, $p = 0.001$, periphery, $p = 0.05$.*, $p < 0.05$; **, $p < 0.01$; ***, $p \leq 0.001$. Data are presented as mean values +/- SEM.

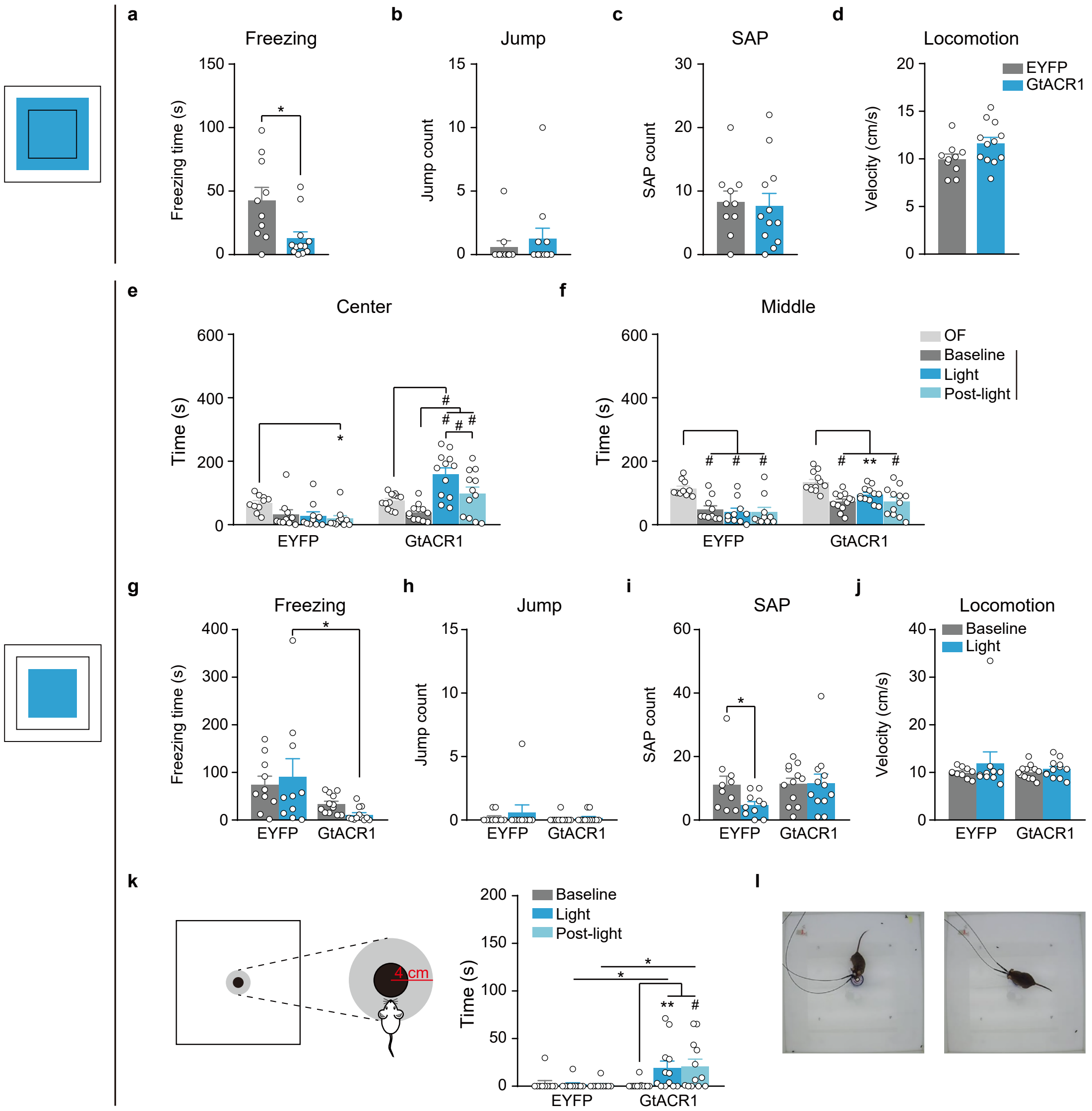

Supplementary Figure 3. The effects of optogenetically inhibiting $\text{AHN}^{\text{Vgat+}}$ neurons on different behaviors and time spent in different zones. (**a-d**) The light was delivered to the center and middle zone. Optogenetic inhibition of $\text{AHN}^{\text{Vgat+}}$ neurons reduced freezing behaviors in GtACR1 males (a, Mann Whitney U test, $p = 0.01$) but did not affect other behaviors such as jump (b, Mann Whitney U test), stretch attended posture (SAP) (c, two-tailed unpaired t test), or locomotion (d, two-tailed unpaired t test). (**e-l**) The light was delivered to the center zone. (e-f) Time spent in the center (e) and middle zone (f) in open field test before or after an object introduction. (g-j) Optogenetic inhibition of $\text{AHN}^{\text{Vgat+}}$ neurons reduced freezing behaviors in GtACR1 males (g) but did not affect other behaviors such as jump (h), SAP (i), or locomotion (j). Two-way repeated measures ANOVA test. (**k-l**) Time spent close up to the object. (k) Left, schematics of the area analyzed. It is a circle with a radius of 4 cm, concentric to the object (battery) which has a radius of ~ 1.8 cm. Right, time spent in the zone before or after an object introduction. Two-way repeated measures ANOVA test. (l) Example video frames showing GtARC1 males got on top of the object during light inhibition. n = 10 EYFP and 12 GtACR1 males. *, $p < 0.05$; **, $p < 0.01$; #, $p < 0.001$. Data are presented as mean values +/- SEM.

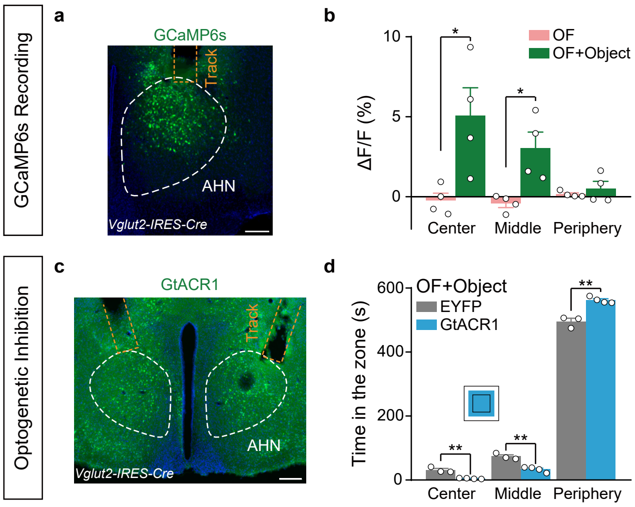

Supplementary Figure 4. Optogenetic inhibition of AHN$^{Vglut2+}$ neurons increases object avoidance.
a-b Fiber photometry recording of AHN$^{Vglut2+}$ neuron response to an object. (a) a representative image showing GCaMP6s expression in AHN$^{Vglut2+}$ neurons and track of the implanted fiber. Scale bar, 200 µm. (b) Average ΔF/F values detected in the "center", "middle" and "periphery" zone of an open field before and after object introduction. n = 4 males. Two-tailed paired t test. center, $p = 0.038$, middle, $p = 0.041$, periphery, $p = 0.456$. **c-d** Optogenetic inhibition of AHN$^{Vglut2+}$ neurons during object approach increased avoidance behavior. (c) a representative image showing GtACR1 expression in AHN$^{Vglut2+}$ neurons and the track of the implanted bilateral fibers. Scale bar, 200 µm. (d) The time spent in the indicated zone after object introduction in EYFP (n = 3) and GtACR1 males (n = 4). Two-tailed unpaired t test. center, $p = 0.002$, middle, $p = 0.002$, periphery, $p = 0.001$. *, $p < 0.05$; **, $p < 0.01$; Data are presented as mean values +/- SEM.

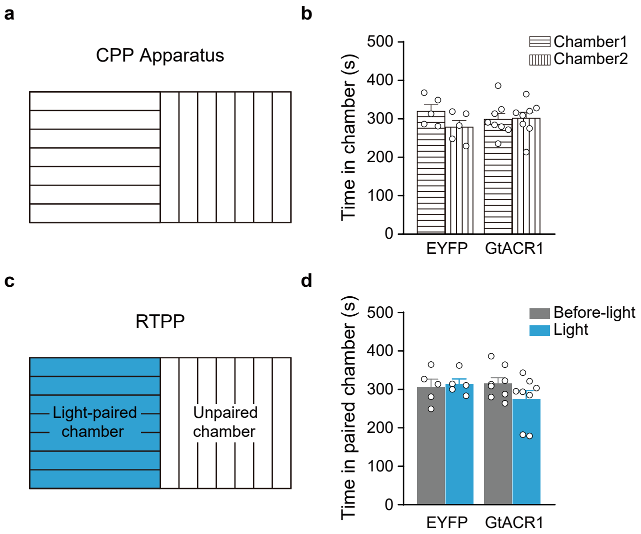

Supplementary Figure 5. Optogenetically inhibiting AHN^{Vgat+} neurons does not lead to place preference.
a Schematics of the apparatus used for testing conditioned place preference (CPP). It has two chambers differing in color/texture. **b** At baseline, EYFP and GtACR1 males spent comparable amount of time spent in either chamber of the apparatus. n = 5 EYFP and 8 GtACR1 males. Two-tailed paired t test. **c** For the experiments, light was randomly delivered whenever the animal entered one of the two chambers. The blue region indicates the light-paired chamber. **d** No differences in time spent in the light-paired chamber before or during light stimulation. n = 5 EYFP and 8 GtACR1 males. Two-tailed paired t test for the EYFP group and Wilcoxon matched-pairs signed rank test for the GtACR1 group. Data are presented as mean values +/- SEM.

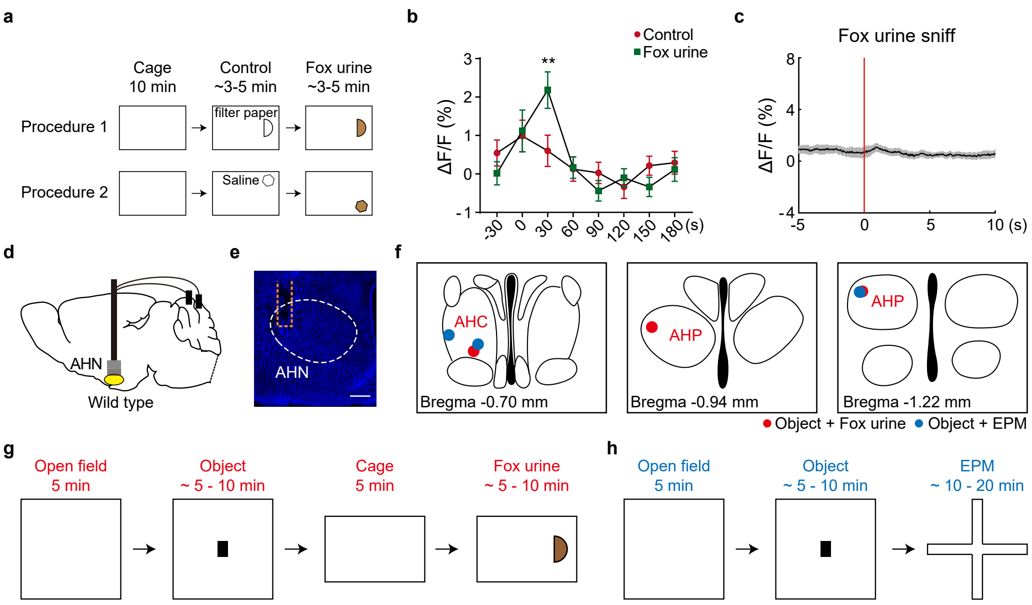

Supplementary Figure 6. Fiber photometry recording of AHN^{Vgat+} neuron response to fox urine and single-unit recording of AHN neurons. **a-c** Fiber photometry recording of AHN^{Vgat+} neuronal response to fox urine exposure. (a) The test procedures. Fox urine was spotted on a piece of filter paper (procedure 1) or onto the cage floor (procedure 2). Signals collected before the introduction of a stimulus (~ 10min) were used as the baseline. (b) Average ΔF/F values for each 30s bin before and after the introduction of fox urine. Controls were presented with a piece of filter paper or with saline spotted onto the cage floor. n = 14 GCaMP6s animals. Two-way repeated measures ANOVA. (c) Average ΔF/F values aligned to the onset of fox urine sniff. n = 7 GCaMP6s males. The solid line indicates the mean and the shaded area is the SEM. **d** Schematics showing electrode implantation in AHN and grounding of the implanted electrodes. **e** A representative *post-hoc* image showing the tip of the implanted electrode lied within AHN. Scale bar, 200 μm. **f** Anatomical tip locations of the implanted electrode in the six recorded mice. Different colored circles represent different experiments as indicated at the bottom. **g-h** Behavioral procedures of single-unit recording experiments. **, p < 0.01. Data are presented as mean values +/- SEM.

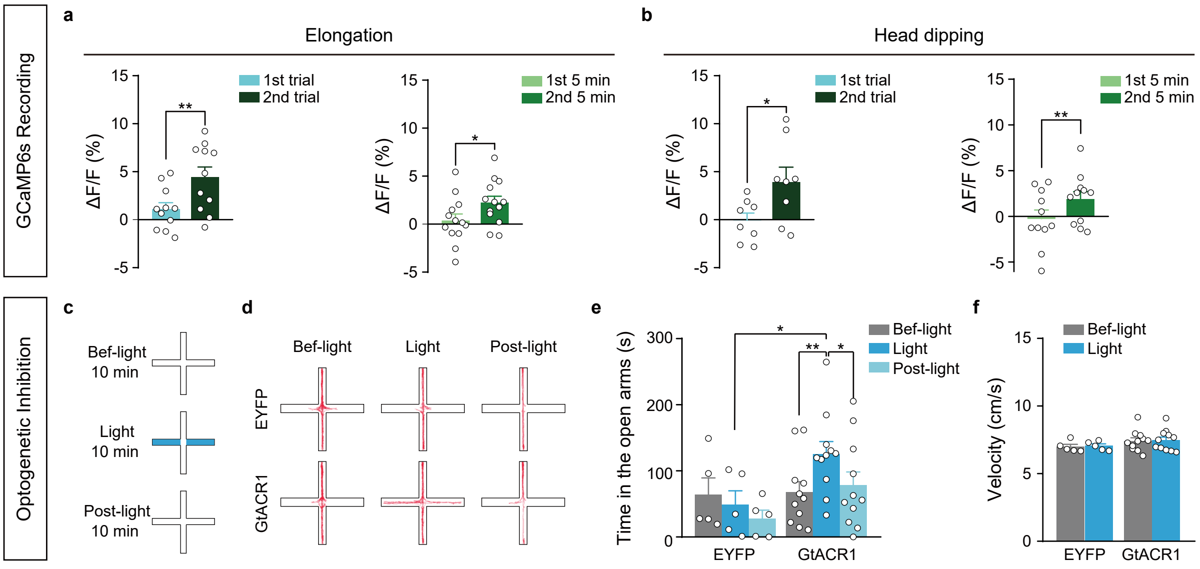

Supplementary Figure 7. Progressive elevation of anxiety-like AHN^{Vgat+} activity on EPM.
a-b Average ΔF/F values during the first and second trial (left) and during the first and second 5 min of the first trial (right) for specific behaviors in EPM open-arm: body elongation (a, left, n = 11, p = 0.003, right, n = 13, p = 0.04) and head dipping (b, left, n = 8, p = 0.01, right, n = 11, p = 0.008). Two-tailed paired t test. **c-f** Optogenetic inhibition of AHN^{Vgat+} neurons reduced open-arm avoidance. (c) Schematics of the light delivery areas and the test procedures (d) Example movement trajectories on EPM from a control EYFP and a GtACR1 male. (e) Time spent in EPM open-arm in before, during, and post-light delivery. Light illumination increased open-arm time in GtACR1 but not control EYFP males. n = 5 EYFP and 11 GtACR1 males. Two-way repeated measures ANOVA. (f) Open-arm velocity. n = 5 EYFP and 11 GtACR1 males. Two-tailed paired t test. *, $p < 0.05$; **, $p < 0.01$. Data are presented as mean values +/- SEM.

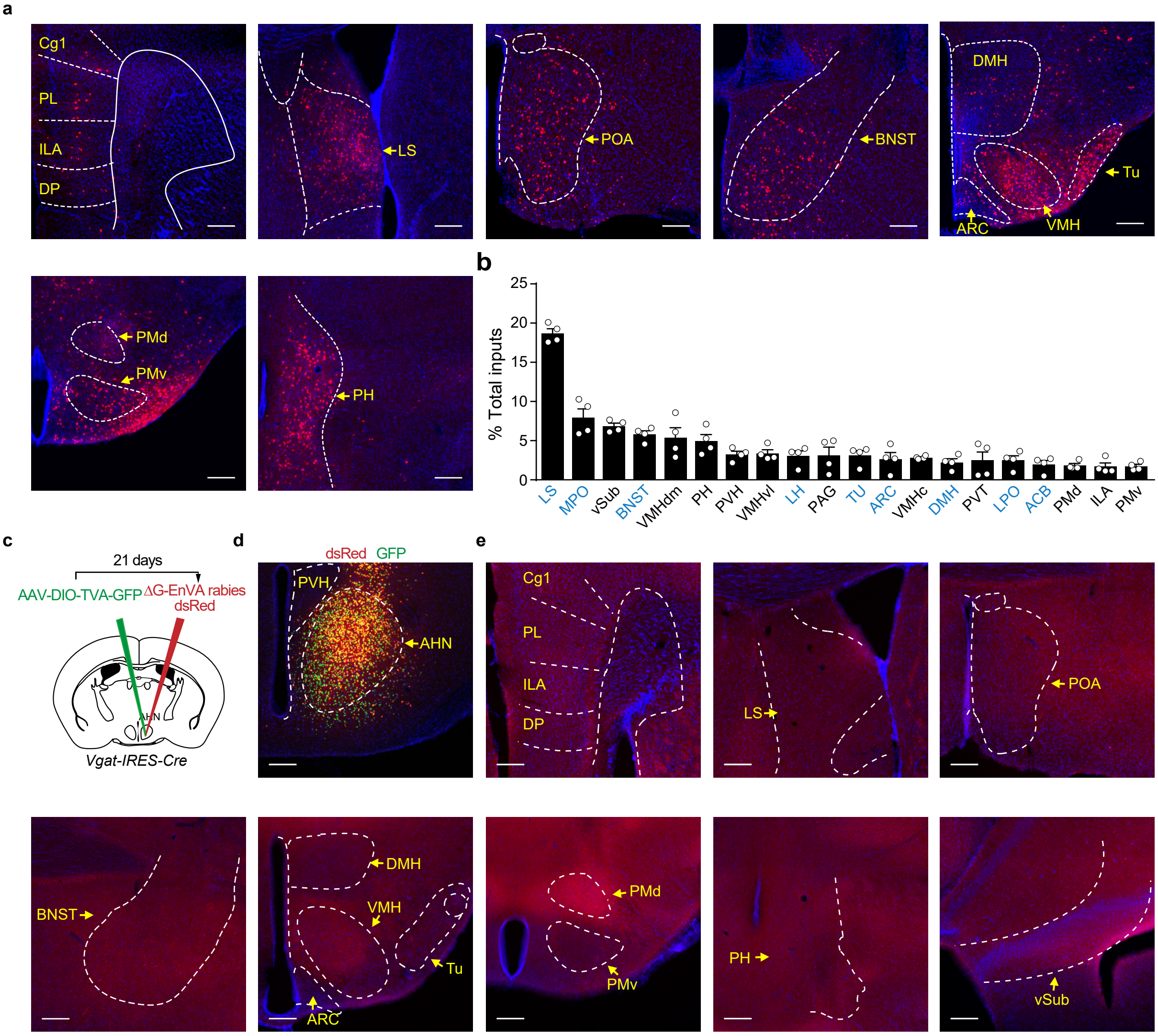

Supplementary Figure 8. Quantification of and control experiments for pseudorabies mediated retrograde tracing of inputs to AHN^{Vgat+} neurons. **a-b** Pseudotyped rabies virus-mediated retrograde tracing of inputs to AHN^{Vgat+} neurons. (a) Representative images showing dsRed+ neurons in areas indicated. Scale bar, 200 µm. (b) Quantification of dsRed+ neurons in each region as % of total dsRed+ cells detected outside of the AHN. n = 4 mice. Light blue text indicates areas consisting of predominantly inhibitory projection neurons (www.mouse.brain-map.org). **c-e** The control experiment. n = 3 mice. (c) Schematics of the viral strategy for the control experiment without RG injection. (d) A representative image showing infection of AHN^{Vgat+} neurons by AAV-DIO-TVA-GFP and EnVA-pseudotyped rabies virus expressing dsRed. Scale bar, 200 µm. (e) Representative images showing no dsRed+ signal in areas indicated. Scale bar, 200 µm. Abbreviations: cingulate cortex area 1 (Cg1), prelimbic area (PL), infralimbic area (ILA), dorsal peduncular area (DP), lateral septum (LS), preoptic area (POA), paraventricular hypothalamic nucleus (PVH), bed nuclei of the stria terminalis (BNST), dorsomedial hypothalamus (DMH), ventromedial hypothalamus (VMH), arcuate hypothalamic nucleus (ARC), tuberal nucleus (TU), dorsal premammillary nucleus (PMd), ventral premammillary nucleus (PMv), posterior hypothalamus (PH), ventral subiculum (vSub). Data are presented as mean values +/- SEM.

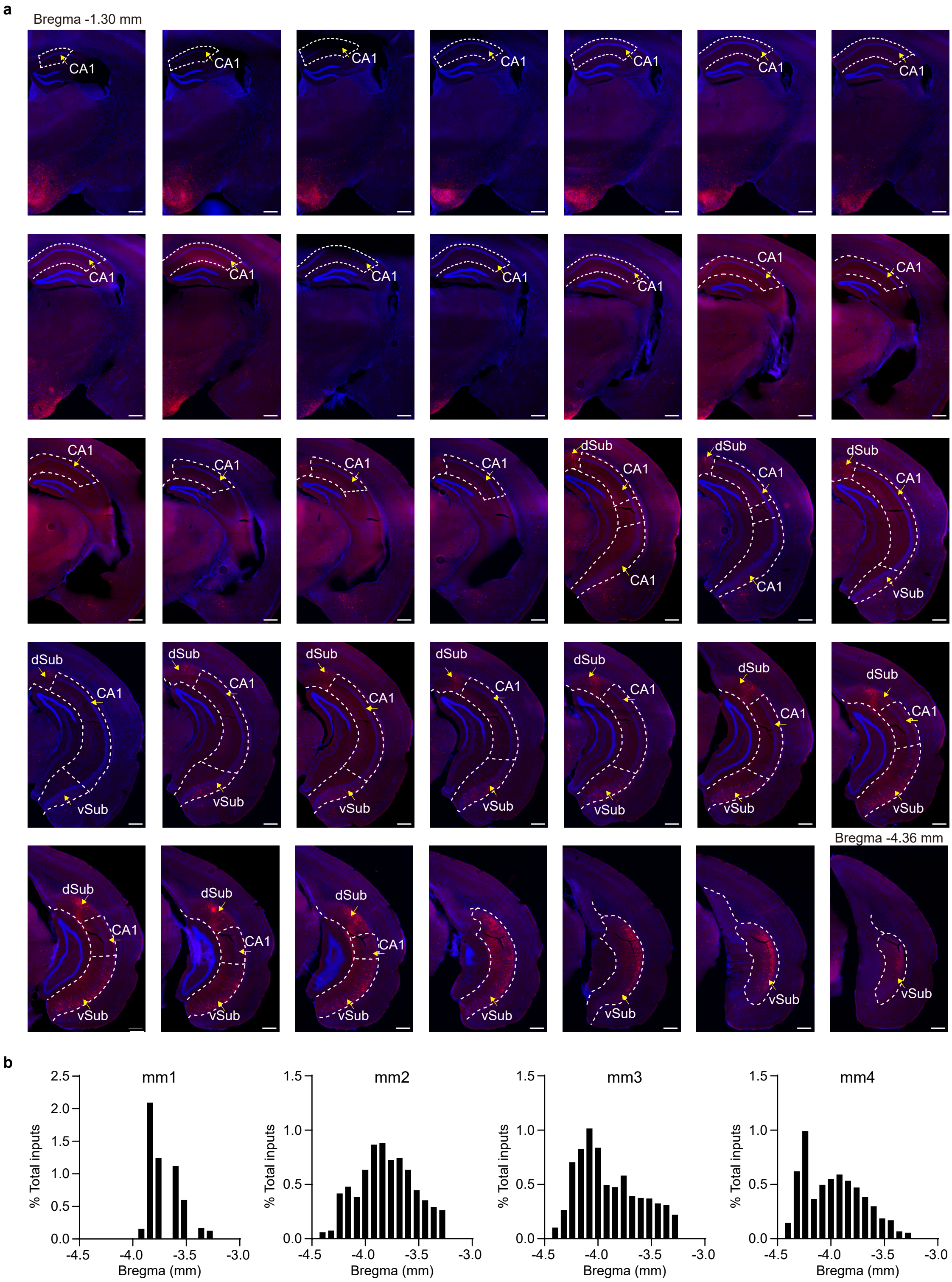

Supplementary Figure 9. Pseudorabies mediated retrograde tracing identifies substantial vSub inputs to AHN^{Vgat+} neurons.
a Montage of brain sections from an example animal depicting the CA1, dSub, and vSub area analyzed. Concentrated input cells were observed in vSub but not CA1 or dSub. Scale bar, 400 µm. **b** Distribution of vSub inputs along the AP axis in individual mice.

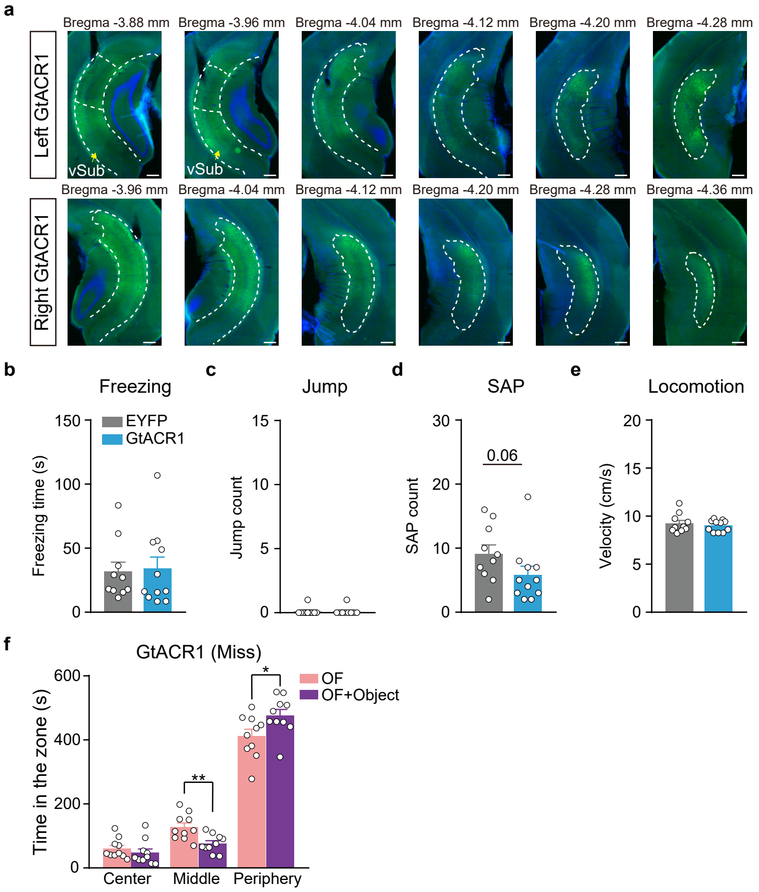

Supplementary Figure 10. The effects of optogenetically inhibiting AHN-projecting vSub neurons on different behaviors in the open field.

a The montage of brain sections from an example animal showing GtACR1 expression in vSub. Scale bar, 300 μm. **b-e** No changes were found for freezing **(b)**, jump **(c)**, and locomotion **(e)**. The trend was that stretch attended posture (SAP) (**d**) was reduced by optogenetic inhibition of AHN-projecting vSub neurons. n = 10 EYFP and 11 GtACR1 males. Mann Whiteny U test. **f** The time spent in the indicated zone before and after object introduction in miss-targeted GtACR1 males (n = 10). In these "miss" animals, light delivery had no effects on object avoidance. Wilcoxon matched-pairs signed rank test for center time, $p = 0.375$, and two-tailed paired t test for middle and periphery time, $p = 0.002$ and 0.044 respectively. *, $p < 0.05$; **, $p < 0.01$. Data are presented as mean values +/- SEM.

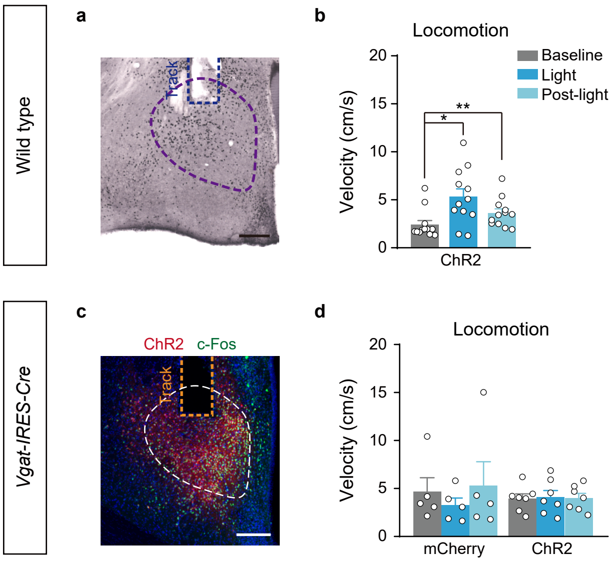

Supplementary Figure 11. The locomotive effects of optogenetic activation of AHN neurons. Pan-neuronal activation of AHN (**a**-**b**) increased locomotion while optogenetic activation of AHN^{Vgat+} neurons (**c**-**d)** was without an effect. One-way repeated measures ANOVA test for (b) and two-way repeated measures ANOVA test for (d). Representative images in (a) & (c) show the track of the implanted fiber and AHN c-Fos signal after light stimulation as analyzed by DAB staining in (a) and fluorescent immunostaining in (c). Scale bar, 200 µm. n = 12 WT ChR2 mice, 5 Vgat-mCherry mice and 7 Vgat-ChR2 mice. *, $p < 0.05$; **, $p < 0.01$. Data are presented as mean values +/- SEM.

Figure	Sample size (n)	Statistical test	p valus
1a	6 mice	Wilcoxon matched-pairs signed rank test<br>Wilcoxon matched-pairs signed rank test<br>Wilcoxon matched-pairs signed rank test	Centrer : p = 0.03 *<br>Middle: p = 0.03 *<br>Periphery: p = 0.03 *
1b	6 mice	Two-tailed paired t test<br>Two-tailed paired t test<br>Two-tailed paired t test	Centrer: p = 0.62<br>Middle: p = 0.84<br>Periphery: p = 1.07
1f	14 mice	Two-tailed paired t test<br>Wilcoxon matched-pairs signed rank test<br>Wilcoxon matched-pairs signed rank test	Centrer: p = 0.0003 ***<br>Middle: p = 0.0004 ***<br>Periphery: p = 0.0009 ***
3d	9 cells	Wilcoxon matched-pairs signed rank test<br>Wilcoxon matched-pairs signed rank test	Baseline vs Light: p = 0.004 **<br>Light vs After light:p = 0.004 **
3g	EYFP 10 mice	Two-tailed paired t test<br>Two-tailed paired t test<br>Two-tailed paired t test	Center: p = 0.04 *<br>Middle: p = 0.002 **<br>Periphery: p = 0.003 **
	GtACR1 12 mice	Wilcoxon matched-pairs signed rank test<br>Two-tailed paired t-test<br>Two-tailed paired t-test	Center: p = 0.18<br>Middle: p = 0.08<br>Periphery: p = 0.63
3j	EYFP 10 mice<br>GtACR1 12 mice	Two-way repeated measures ANOVA<br>Factor1: Phase (OF, Baseline, Light, Post-light)<br>Factor2: Virus (EYFP, GtACR1)<br>Tukey's multiple comparisons test	Phase: F(3, 60) = 15.9 ,p < 0.0001 ***<br>Virus:F(1,20) = 14.61, p = 0.0011**<br>Interaction: F(3,60) = 11.1, p <0.0001 ***<br>Multiple comparisons<br>EYFP:<br>OF vs Baseline: p = 0.0004 ***<br>OF vs Light: p < 0.0001 ***<br>OF vs Post-light: p < 0.0001 ***<br>Baseline vs Light: p = 0.95<br>Baseline vs Post-light: p = 0.80<br>Light vs Post-light: p = 0.98<br>GtACR1:<br>OF vs Baseline: p = 0.0001 ***<br>OF vs Light: p = 0.19<br>OF vs Post-light: p = 0.29<br>Baseline vs Light: p < 0.0001 ***<br>Baseline vs Post-light: p = 0.035*<br>Light vs Post-light: p = 0.002**

Figure	Sample size (n)	Statistical test	p valus
4g	13 units	Wilcoxon matched-pairs signed rank test<br>Wilcoxon matched-pairs signed rank test<br>Wilcoxon matched-pairs signed rank test	Object approach: p = 0.03 *<br>Fox urine in: p = 0.0002 ***<br>Fox urine sniff: p = 0.31
4h	9 units	Two-tailed paired t test<br>Two-tailed paired t test<br>Two-tailed paired t test	Object approach: p = 0.006 **<br>Fox urine in: p = 0.70<br>Fox urine sniff: p = 0.08
5a	13 mice	Two-tailed paired t test	Open-arm vs Closed arm: p = 0.048 *
5b	11 mice	Wilcoxon matched-pairs signed rank test	1st trial vs 2nd trial: p = 0.01 *
5c	11 mice	Two-tailed paired t test	1st trial vs 2nd trial: p = 0.006 **
5d	12 mice	Wilcoxon matched-pairs signed rank test	1st 5 min vs 2nd 5 min: p = 0.077
5j	EYFP 7 mice<br>GtACR1 7 mice	Wilcoxon matched-pairs signed rank test<br>Two-tailed paired t test	EYFP: p = 0.69<br>GtACR1: p = 0.003 **
5k	7 mice	Two-way repeated measures ANOVA<br>Factor1: Time (1st 5 min, 2nd 5 min)<br>Factor2: Arm (Non-paired, Light-paired)<br>Sidak's multiple comparisons test	Time: F(1, 12) = 6.132 , p = 0.03*<br>Arm: F(1,12) = 9.462, p = 0.01**<br>Interaction: F(1,12) = 0.537, p = 0.48<br>Multiple comparisons<br>1st 5 min:<br>Non-paired vs Light-paired, p = 0.14<br>2nd 5 min:<br>Non-paired vs Light-paired, p = 0.02*
5l	7 mice	Two-tailed paired t test	Non-paired open-arm vs Light-paired open-arm: p = 0.27
6m	8 mice	Two-way repeated measures ANOVA<br>Factor1: Time (1st 5 min, 2nd 5 min)<br>Factor2: Arm (Open-arm, Closed arm)<br>Sidak's multiple comparisons test	Time: F(1, 14) =17.28 , p = 0.001***<br>Arm: F(1,14) = 58.03, p < 0.0001***<br>Interaction: F(1,14) = 6.089, p = 0.03*<br>Multiple comparisons<br>1st 5 min:<br>Open-arm vs Closed arm: p < 0.0001***<br>2nd 5 min:<br>Open-arm vs Closed arm: p < 0.0001***<br>Open-arm:

Figure	Sample size (n)	Statistical test	p valus
			1st 5 min vs 2nd 5min: p = 0.0007 ***
			Closed arm:
			1st 5 min vs 2nd 5min: p = 0.44
7g	EYFP 10 mice	Two-tailed paired t test	Center: p = 0.24
		Two-tailed paired t test	Middle: p < 0.0001 ***
		Two-tailed paired t test	Periphery: p = 0.002 **
7h	GtACR1 11 mice	Wilcoxon matched-pairs signed rank test	Center: p = 0.04 *
		Two-tailed paired t test	Middle: p = 0.006 **
		Wilcoxon matched-pairs signed rank test	Periphery: p = 0.32
7j	EYFP 10 mice	Wilcoxon matched-pairs signed rank test	EYFP: p = 0.77
	GtACR1 9 mice	Two-tailed paired t test	GtACR1: p = 0.03 *
7k	9 mice	Wilcoxon matched-pairs signed rank test	Non-paired open-arm vs Light-paired open-arm: p = 0.16
S.1b	8 mice	Wilcoxon matched-pairs signed rank test	Center: p = 0.46
		Two-tailed paired t test	Middle: p = 0.54
		Wilcoxon matched-pairs signed rank test	Periphery: p = 0.84
S.1f	10 mice	Two-tailed paired t test	Center: p < 0.0001 ***
		Two-tailed paired t test	Middle: p = 0.86
		Two-tailed paired t test	Periphery: p < 0.0001 ***
			Battery:
S.2c	16 mice	Wilcoxon matched-pairs signed rank test	Center: p = 0.67
		Two-tailed paired t test	Middle: p < 0.0001 ***
		Wilcoxon matched-pairs signed rank test	Periphery: p = 0.005 **
			Cuboid:
		Wilcoxon matched-pairs signed rank test	Center: p = 0.18
		Two-tailed paired t test	Middle: p = 0.0004 ***
		Two-tailed paired t test	Periphery: p = 0.02 *
			Toy:
		Wilcoxon matched-pairs signed rank test	Center: p = 0.04 *
		Wilcoxon matched-pairs signed rank test	Middle: p < 0.0001 ***
		Wilcoxon matched-pairs signed rank test	Periphery: p = 0.005 **
			Clip
		Two-tailed paired t test	Center: p = 0.75

Figure	Sample size (n)	Statistical test	p valus
		Two-tailed paired t test<br>Two-tailed paired t test	Middle: p = 0.001 ***<br>Periphery: p = 0.051
S.3a	EYFP 10 mice<br>GtACR1 12 mice	Mann Whitney U test	EYFP vs GtACR1: p = 0.01 *
S.3b	EYFP 10 mice<br>GtACR1 12 mice	Mann Whitney U test	EYFP vs GtACR1: p = 0.53
S.3c	EYFP 10 mice<br>GtACR1 12 mice	Two-tailed unpaired t test	EYFP vs GtACR1: p = 0.82
S.3d	EYFP 10 mice<br>GtACR1 12 mice	Two-tailed unpaired t test	EYFP vs GtACR1: p = 0.07
S.3e	EYFP 10 mice<br>GtACR1 12 mice	Two-way repeated measures ANOVA<br>Factor1: Phase (OF, Baseline, Light, Post-light)<br>Factor2: Virus (EYFP, GtACR1)<br>Tukey's multiple comparisons test	Phase: F(3, 60) = 9.966, p < 0.0001 ***<br>Virus:F(1,20) = 14.24, p = 0.0012 **<br>Interaction: F(3,60) = 15.38, p < 0.0001 ***<br>Multiple comparisons<br>EYFP:<br>OF vs Baseline: p = 0.13<br>OF vs Light: p = 0.07<br>OF vs Post-light: p = 0.02 *<br>Baseline vs Light: p = 0.99<br>Baseline vs Post-light: p = 0.84<br>Light vs Post-light: p = 0.95<br>GtACR1:<br>OF vs Baseline: p = 0.06<br>OF vs Light: p < 0.0001 ***<br>OF vs Post-light: p = 0.45<br>Baseline vs Light: p < 0.0001 ***<br>Baseline vs Post-light: p = 0.0009 ***<br>Light vs Post-light: p = 0.0006 ***
S.3f	EYFP 10 mice<br>GtACR1 12 mice	Two-way repeated measures ANOVA<br>Factor1: Phase (OF, Baseline, Light, Post-light)<br>Factor2: Virus (EYFP, GtACR1)<br>Tukey's multiple comparisons test	Phase: F(3, 60) = 27.96, p < 0.0001 ***<br>Virus:F(1,20) = 9.115, p = 0.007 **<br>Interaction: F(3,60) = 1.68, p = 0.18<br>Multiple comparisons<br>EYFP:

Figure	Sample size (n)	Statistical test	p valus
			OF vs Baseline: $p < 0.0001$ ***<br>OF vs Light: $p < 0.0001$ ***<br>OF vs Post-light: $p < 0.0001$ ***<br>Baseline vs Light: $p = 0.91$<br>Baseline vs Post-light: $p = 0.91$<br>Light vs Post-light: $p > 0.9999$<br>GtACR1:<br>OF vs Baseline: $p < 0.0001$ ***<br>OF vs Light: $p = 0.0055$ **<br>OF vs Post-light: $p < 0.0001$ ***<br>Baseline vs Light: $p = 0.23$<br>Baseline vs Post-light: $p = 0.9998$<br>Light vs Post-light: $p = 0.27$
S.3g	EYFP 10 mice<br>GtACR1 12 mice	Two-way repeated measures ANOVA<br>Factor1: Phase (Baseline, Light)<br>Factor2: Virus (EYFP, GtACR1)<br>Sidak's multiple comparisons test	Phase: $F(1, 20) = 0.05$, $p = 0.83$<br>Virus:$F(1,20) = 6.227$, $p = 0.02$ *<br>Interaction: $F(1,20) = 2.664$, $p = 0.12$<br>Multiple comparisons<br>Baseline<br>EYFP vs GtACR1: $P = 0.26$<br>Light<br>EYFP vs GtACR1: $P = 0.01$ *<br>EYFP<br>Baseline vs Light: $p = 0.58$<br>GtACR1<br>Baseline vs Light: $p = 0.33$
S.3h	EYFP 10 mice<br>GtACR1 12 mice	Two-way repeated measures ANOVA<br>Factor1: Phase (Baseline, Light)<br>Factor2: Virus (EYFP, GtACR1)<br>Sidak's multiple comparisons test	Phase: $F(1, 20) = 1.007$, $p = 0.33$<br>Virus:$F(1,20) = 0.69$, $p = 0.42$<br>Interaction: $F(1,20) = 0.43$, $p = 0.52$<br>Multiple comparisons<br>Baseline<br>EYFP vs GtACR1: $P = 0.95$<br>Light<br>EYFP vs GtACR1: $P = 0.50$<br>EYFP<br>Baseline vs Light: $p = 0.47$<br>GtACR1<br>Baseline vs Light: $p = 0.96$
S.3i	EYFP 10 mice<br>GtACR1 12 mice	Two-way repeated measures ANOVA<br>Factor1: Phase (Baseline, Light)<br>Factor2: Virus (EYFP, GtACR1)	Phase: $F(1, 20) = 3.306$, $p = 0.08$<br>Virus:$F(1,20) = 1.677$, $p = 0.21$<br>Interaction: $F(1,20) = 3.669$, $p = 0.07$

Figure	Sample size (n)	Statistical test	p valus
		Sidak's multiple comparisons test	Multiple comparisons<br>Baseline<br>EYFP vs GtACR1: P = 0.99<br>Light<br>EYFP vs GtACR1: P = 0.08<br>EYFP<br>Baseline vs Light: p = 0.04 *<br>GtACR1<br>Baseline vs Light: p = 0.997
S.3j	EYFP 10 mice<br>GtACR1 12 mice	Two-way repeated measures ANOVA<br>Factor1: Phase (Baseline, Light)<br>Factor2: Virus (EYFP, GtACR1)<br>Sidak's multiple comparisons test	Phase: F(1, 20) = 1.304, p = 0.27<br>Virus:F(1,20) = 0.2022, p = 0.66<br>Interaction: F(1,20) = 0.3448, p = 0.56<br>Multiple comparisons<br>Baseline<br>EYFP vs GtACR1: P = 0.998<br>Light<br>EYFP vs GtACR1: P = 0.72<br>EYFP<br>Baseline vs Light: p = 0.45<br>GtACR1<br>Baseline vs Light: p = 0.90
S.3k	EYFP 10 mice<br>GtACR1 12 mice	Two-way repeated measures ANOVA<br>Factor1: Phase (Baseline, Light, Post-light)<br>Factor2: Virus (EYFP, GtACR1)<br>Sidak's multiple comparisons test<br><br><br><br><br>Tukey's multiple comparisons test	Phase: F(2, 40) = 3.896, p = 0.03 *<br>Virus:F(1,20) = 4.48, p = 0.047 *<br>Interaction: F(2,40) = 5.26, p = 0.009 **<br>Multiple comparisons<br>EYFP vs GtACR<br>Baseline: p = 0.99<br>Light: p = 0.045 *<br>Post-light: p = 0.02 *<br>Multiple comparisons<br>EYFP<br>Baseline vs Light: p = 0.97<br>Baseline vs Post-light: p = 0.95<br>Light vs Post-light: p = 0.996<br>GtACR1<br>Baseline vs Light: p = 0.002 **<br>Baseline vs Post-light: p = 0.0006 ***<br>Light vs Post-light: p = 0.92

Figure	Sample size (n)	Statistical test	p valus
S.4b	4 mice	Two-tailed paired t test<br>Two-tailed paired t test<br>Two-tailed paired t test	Centrer: $p = 0.038$ *<br>Middle: $p = 0.041$ *<br>Periphery: $p = 0.456$
S.4d	EYFP 3 mice<br>GtACR1 4 mice	Two-tailed unpaired t test<br>Two-tailed unpaired t test<br>Two-tailed unpaired t test	Center: $p = 0.002$ **<br>Middle: $p = 0.002$ **<br>Periphery: $p = 0.001$ **
S.5b	EYFP 5 mice<br>GtACR1 8 mice	Two-tailed paired t test<br>Two-tailed paired t test	EYFP: $p = 0.30$<br>GtACR1: $p = 0.92$
S.5d	EYFP 5 mice<br>GtACR1 8 mice	Two-tailed paired t test<br>Wilcoxon matched-pairs signed rank test	EYFP: $p = 0.53$<br>GtACR1: $p = 0.11$
S.6b	14 mice	Two-way repeated measures ANOVA<br>Factor1: Time (-30s, 0s, 30s, 60s, 90s, 120s, 150s, 180s)<br>Factor2: Manipulation (Control, Fox urine)<br>Sidak's multiple comparisons test	Time: $F(7, 182) = 6.558$, $p < 0.0001$ ***<br>Manipulation: $F(1,26) = 0.025$, $p = 0.88$<br>Interaction: $F(7, 182) = 2.273$, $p = 0.03$ *<br>Multiple comparisons90s<br>Control vs Fox urine<br>-30s: $p = 0.92$<br>0s: $p > 0.9999$<br>30s: $p = 0.009$ **<br>60s: $p > 0.9999$<br>90s: $p = 0.96$<br>120s: $p = 0.9996$<br>150s: $p = 0.90$<br>180s: $p > 0.9999$
S.7a	11 mice<br>13 mice	Two-tailed paired t test<br>Two-tailed paired t test	1st trial vs 2nd trial: $p = 0.003$ **<br>1st 5 min vs 2nd 5 min: $p = 0.04$ *
S.7b	8 mice<br>11 mice	Two-tailed paired t test<br>Two-tailed paired t test	1st trial vs 2nd trial: $p = 0.01$ *<br>1st 5 min vs 2nd 5 min: $p = 0.008$ **
S.7e	EYFP 5 mice<br>GtACR1 11 mice	Two-way repeated measures ANOVA<br>Factor1: Phase (Bef-light, Light, Post-light)<br>Factor2: Virus (EYFP, GtACR1)<br>Tukey's multiple comparisons test	Phase: $F(2, 28) = 3.21$, $p = 0.056$<br>Virus:$F(1,14) = 2.752$, $p = 0.12$<br>Interaction: $F(2,28) = 3.669$, $p = 0.04$ *<br>Multiple comparisons<br>EYFP

Figure	Sample size (n)	Statistical test	p valus
			Bef-light vs Light: p = 0.79<br>Bef-light vs Post-light: p = 0.27<br>Light vs Post-light: p = 0.63<br>GtACR1<br>Bef-light vs Light: p = 0.002 **<br>Bef-light vs Post-light: p = 0.776<br>Light vs Post-light: p = 0.01 *
		Sidak's multiple comparisons test	Multiple comparisons<br>EYFP vs GtAVR1<br>Bef-light: p = 0.999<br>Light: p = 0.047 *<br>Post-light: p = 0.29
S.7f	EYFP 5 mice<br>GtACR1 11 mice	Two-tailed paired t test<br>Two-tailed paired t test	EYFP: p = 0.75<br>GtACR1: P = 0.64
S.10b	EYFP 10 mice<br>GtACR1 11 mice	Mann Whitney U test	EYFP vs GtAVR1: p = 0.62
S.10c	EYFP 10 mice<br>GtACR1 11 mice	Mann Whitney U test	EYFP vs GtAVR1: p > 0.9999
S.10d	EYFP 10 mice<br>GtACR1 11 mice	Mann Whitney U test	EYFP vs GtAVR1: p = 0.06
S.10e	EYFP 10 mice<br>GtACR1 11 mice	Mann Whitney U test	EYFP vs GtAVR1: p = 0.76
S.10f	10 mice	Wilcoxon matched-pairs signed rank test<br>Two-tailed paired t test<br>Two-tailed paired t test	Center: p = 0.375<br>Middle: p = 0.002 **<br>Periphery: p = 0.044 *
S.11b	12 mice	One-way repeated measures ANOVA<br>Factor: Phase (Baselinet, Light, Post-light)	Phase: F(1.194, 13.14) = 10.69,<br>p = 0.005 **
		Tukey's multiple comparisons test	Multiple comparisons<br>Baselinet vs Light: p = 0.01 *<br>Baseline vs Post-light: p = 0.007 **<br>Light vs Post-light: p = 0.06
S.11d	mCherry 5 mice<br>ChR2 7 mice	Two-way repeated measures ANOVA<br>Factor1: Phase (Baseline, Light, Post-light)	Phase: F(2, 20) = 1.49, p = 0.25<br>Virus:F(1,10) = 0.076, p = 0.79<br>Interaction: F(2,20) = 1.938, p = 0.17

Figure	Sample size (n)	Statistical test	p valus
		Factor2: Virus (mCherry, ChR2)<br>Tukey's multiple comparisons test<br><br><br><br><br><br><br><br><br>Sidak's multiple comparisons test	Multiple comparisons<br>mCherry<br>Baseline vs Light: p = 0.27<br>Baseline vs Post-light: p = 0.73<br>Light vs Post-light: p = 0.07<br>ChR2<br>Baseline vs Light: p = 0.97<br>Baseline vs Post-light: p = 0.998<br>Light vs Post-light: p = 0.99<br><br>mCherry vs ChR2<br>Baseline: p = 0.96<br>Light: p = 0.93<br>Post-light: p = 0.79

Table 1. Summary of statistical analyses.
