# Supplementary material for: A circuit from the ventral subiculum to anterior hypothalamic nucleus GABAergic neurons essential for anxiety-like behavioral avoidance
Source: Nat Commun. 2022 Dec 3;13:7464. doi: 10.1038/s41467-022-35211-7 (PMC9719513; doi:10.1038/s41467-022-35211-7)
Supplement: Supplementary file 3 — Reporting Summary [file 41467_2022_35211_MOESM3_ESM.pdf]

nature portfolio

Corresponding author(s): Xiaohong Xu

Last updated by author(s): Oct 16, 2022

Reporting Summary

Nature Portfolio wishes to improve the reproducibility of the work that we publish. This form provides structure for consistency and transparency in reporting. For further information on Nature Portfolio policies, see our Editorial Policies and the Editorial Policy Checklist.

Statistics

For all statistical analyses, confirm that the following items are present in the figure legend, table legend, main text, or Methods section.

n/a	Confirmed	
☐	☒	The exact sample size (n) for each experimental group/condition, given as a discrete number and unit of measurement
☐	☒	A statement on whether measurements were taken from distinct samples or whether the same sample was measured repeatedly
☐	☒	The statistical test(s) used AND whether they are one- or two-sided<br>*Only common tests should be described solely by name; describe more complex techniques in the Methods section.*
☒	☐	A description of all covariates tested
☐	☒	A description of any assumptions or corrections, such as tests of normality and adjustment for multiple comparisons
☐	☒	A full description of the statistical parameters including central tendency (e.g. means) or other basic estimates (e.g. regression coefficient) AND variation (e.g. standard deviation) or associated estimates of uncertainty (e.g. confidence intervals)
☐	☒	For null hypothesis testing, the test statistic (e.g. F, t, r) with confidence intervals, effect sizes, degrees of freedom and P value noted<br>*Give P values as exact values whenever suitable.*
☒	☐	For Bayesian analysis, information on the choice of priors and Markov chain Monte Carlo settings
☒	☐	For hierarchical and complex designs, identification of the appropriate level for tests and full reporting of outcomes
☐	☒	Estimates of effect sizes (e.g. Cohen's d, Pearson's r), indicating how they were calculated

Our web collection on statistics for biologists contains articles on many of the points above.

Software and code

Policy information about availability of computer code

Data collection	Fiber photometry: commercialized software from Biolink Optics Technology<br>Whole cell recording: commercialized software Clampex 10.2 from Molecular Devices<br>Single unit recording: commercialized software, NeuroPhys Acquisition System from Neurosys2.8.0.8, USA and NeuroLego System from Jiangsu Brain Medical Technology Co.ltd<br>Behavior data: EthoVision XT
Data analysis	MatLab 2009a<br>MatLab 2014b<br>MClust 4.4<br>ImageJ 1.48<br>GraphPad Prism 7<br>EthoVision XT<br>Custom code to analyze fiber photomery and single unit recording data which is available upon request<br>https://github.com/xulab2022/AHN

For manuscripts utilizing custom algorithms or software that are central to the research but not yet described in published literature, software must be made available to editors and reviewers. We strongly encourage code deposition in a community repository (e.g. GitHub). See the Nature Portfolio guidelines for submitting code & software for further information.

Data

Policy information about availability of data

All manuscripts must include a data availability statement. This statement should provide the following information, where applicable:

- Accession codes, unique identifiers, or web links for publicly available datasets
- A description of any restrictions on data availability
- For clinical datasets or third party data, please ensure that the statement adheres to our policy

Source data are provided with this paper and publicly available at https://github.com/xulab2022/AHN and http://atlas.brain-map.org/

Human research participants

Policy information about studies involving human research participants and Sex and Gender in Research.

Reporting on sex and gender	n/a
Population characteristics	n/a
Recruitment	n/a
Ethics oversight	n/a

Note that full information on the approval of the study protocol must also be provided in the manuscript.

Field-specific reporting

Please select the one below that is the best fit for your research. If you are not sure, read the appropriate sections before making your selection.

[x] Life sciences [ ] Behavioural & social sciences [ ] Ecological, evolutionary & environmental sciences

For a reference copy of the document with all sections, see nature.com/documents/nr-reporting-summary-flat.pdf

Life sciences study design

All studies must disclose on these points even when the disclosure is negative.

Sample size	No sample size calculation was performed. The sample size were similar to those reported in previous publications, PubMed 34980925, PubMed 27641503 and PubMed 29397273.
Data exclusions	MIce that were mis-hits for viral injection or optic fiber placement were excluded from further analysis unless noted in the text.
Replication	All attempts at replication were successful. Experiments and data analysis were done by different experimenters blindly. And all data related behavior were collected from more than one batch mice. And all the histological experiments and images were collected from at least 3 mice, except for 2 mice in fig 4c fox urine-object group and at least two batches except for verification of Vgat-IRES-Cre mouse line, the ratios of vgat+ and vglut2+ neurons in AHN and c-fos staining in fig 4a-c.
Randomization	All animal were randomly assigned to different groups
Blinding	The investigators were blinded to group allocation during data collection and analysis in fiber photometry and optogenetic experiments. WT mice tested in OF+Object (fig 1a), OF (fig 1b) and in c-fos staining (fig 4a-c) were only analyzed blindly, because group allocation was clear during data collection.

Reporting for specific materials, systems and methods

We require information from authors about some types of materials, experimental systems and methods used in many studies. Here, indicate whether each material, system or method listed is relevant to your study. If you are not sure if a list item applies to your research, read the appropriate section before selecting a response.

Materials & experimental systems

n/a	Involved in the study
☐	☒ Antibodies
☒	☐ Eukaryotic cell lines
☒	☐ Palaeontology and archaeology
☐	☒ Animals and other organisms
☒	☐ Clinical data
☒	☐ Dual use research of concern

Methods

n/a	Involved in the study
☒	☐ ChIP-seq
☒	☐ Flow cytometry
☒	☐ MRI-based neuroimaging

Antibodies

Antibodies used

Chicken anti-GFP: Abcam, Cat# ab13970, dilution 1:300
Goat anti-chicken, Alexa Fluor 488: Jackson Immuno Research Laboratories, Cat# 103-545-155, dilution 1:300
Guinea pig anti-c-Fos : Synaptic Systems, Cat #226004, dilution 1:1000
Rabbit anti-c-Fos: Synaptic Systems, Cat #226003, dilution 1:20000
Goat-anti-guinea pig Alexa Fluor 647 : Jackson Immuno Research Laboratories, Cat #106-605-003, dilution 1:1000
Biotin-conjugated goat-anti-rabbit secondary antibody : Jackson Immuno Research Laboratories, Cat #111-065-003, dilution 1:1000

Validation

Chicken anti-GFP: RRID:AB_300798
Goat anti-chicken: RRID:AB_2337390
Guinea pig anti-c-Fos : RRID:AB_2619946
Rabbit anti-c-Fos: RRID:AB_2231974
Goat-anti-guinea pig Alexa Fluor 647 : RRID:AB_2337446
Biotin-conjugated goat-anti-rabbit secondary antibody: RRID:AB_2337959

Animals and other research organisms

Policy information about studies involving animals; ARRIVE guidelines recommended for reporting animal research, and Sex and Gender in Research

Laboratory animals

All animals used in the study were adult males aged between 8-30 weeks, including C57BL/6J, Vgat-IRES-Cre and Vglut2-IRES-Cre. The animals were housed with ad libitum food and water under a reversed 12:12 hr light-dark cycle with temperature controlled between 21 and 23 °C and humidity-controlled between 40–70%.

Wild animals

No wild animals were used

Reporting on sex

only male mice were used

Field-collected samples

No field-collected samples were used

Ethics oversight

Experiment protocols were approved by the Animal Care and Use Committee of the Institute of Neuroscience, Chinese Academy of Sciences, Shanghai, China (IACUC No. NA-01602016) or by the Hubei Provincial Animal Care and Use Committee and the Animal Experimentation Ethics Committee of Huazhong University of Science and Technology (IACUC No.844F) or by the Administrative Panel on Laboratory Animal Care at the National Institute of Biological Sciences.

Note that full information on the approval of the study protocol must also be provided in the manuscript.
